# Safety and Immunogenicity of a Heterologous Booster of Protein Subunit Vaccine MVC-COV1901 after Two Doses of Adenoviral Vector Vaccine AZD1222

**DOI:** 10.3390/vaccines10101701

**Published:** 2022-10-11

**Authors:** Shu-Hsing Cheng, Yi-Chun Lin, Cheng-Pin Chen, Chien-Yu Cheng

**Affiliations:** 1 Department of Infectious Diseases, Taoyuan General Hospital, Ministry of Health and Welfare, Taoyuan 330, Taiwan; 2 School of Public Health, Taipei Medical University, Taipei 110, Taiwan; 3 Institute of Public Health, School of Medicine, National Yang-Ming Chiao Tung University, Taipei 112, Taiwan; 4 School of Clinical Medicine, National Yang-Ming Chiao Tung University, Taipei 112, Taiwan

**Keywords:** COVID-19 vaccine, SARS-CoV-2, heterologous booster, protein subunit vaccine, MVC-COV1901, AZD1222

## Abstract

We report the safety and immunogenicity results in participants administrated with a booster dose of protein subunit vaccine MVC-COV1901 at 12 (Group A) or 24 (Group B) weeks after two doses of AZD1222 (ChAdOx1 nCoV-19). The administration of the MVC-COV1901 vaccine as a booster dose in both groups was generally safe. There were no serious adverse events related to the intervention as adverse events reported were “mild” or “moderate” in nature. In subjects fully vaccinated with two doses of AZD1222, waning antibody immunity was apparent within six months of the second dose of AZD1222. At one month after the MVC-COV1901 booster dose, those who were vaccinated within 12 weeks after the last AZD1222 dose (Group A) had anti-SARS-CoV-2 spike IgG antibody titers and neutralizing antibody titers which were 14- and 6.5-fold increased, respectively, when compared to the titer levels on the day of the booster dose. On the other hand, fold-increase a month post-booster in people who had a booster 24 weeks after the last AZD1222 dose (Group B) were 19.5 and 14.0 times for anti-SARS-CoV-2 spike IgG antibody titers and neutralizing antibody titers, respectively. Among those who were vaccinated within 12 weeks after the last AZD1222 dose, we also observed 5.2- and 5.6-fold increases in neutralizing titer levels against ancestral strain and Omicron variant pseudovirus after the booster dose, respectively. These results support the use of MVC-COV1901 as a heterologous booster for individuals vaccinated with AZD1222. Furthermore, regardless of the dosing schedule, the combination of AZD1222 primary series and MVC-COV1901 booster can be cost-effective and suitably applied to low- and middle-income countries (LMIC).

## 1. Introduction

The COVID-19 pandemic is claiming millions of lives worldwide, and the negative economic and public health impacts may have been far greater without the currently approved vaccines, including the two mRNA- and two adenovirus-based anti-SARS-CoV-2 vaccines from Moderna or Pfizer/BioNTech and Oxford/AstraZeneca or Johnson and Johnson, respectively. These vaccines are highly efficacious in preventing severe disease, hospitalization and death and have demonstrated good safety records overall [1]. Nevertheless, as with all vaccines, there are rare but severe safety signals that have led to limited applicability to specific patient subgroups. Examples are the mRNA vaccines associated with rare events of myocarditis in younger males and thrombosis related to adenoviral vector vaccines [2,3].

New evidence shows challenges with increased infectiousness and immune evasion of emerging variants of concern (VCs), including Delta and Omicron variants, and the discovery of a relatively short duration of protection conferred by COVID-19 vaccines [4,5]. These concerns developed with the resurgence of cases and the observation that vaccinated people show increasing rates of infection starting about 6 months post-vaccination [6]. Thus, the consensus is building that long-term COVID-19 control may be achieved by booster shots that may become annual events [7].

AstraZeneca AZD1222 has the lowest cost amongst approved vaccines. Since there is no need for an extreme-cold chain infrastructure such as the one needed for the mRNA vaccines, it is ideal for use in lower and middle-income countries [8,9]. However, AZD1222 is of weaker immunogenicity than other widely used mRNA vaccines, but boosters with different vaccines (heterologous boosting) may compensate for this potential deficiency, as shown in the COV-BOOST trial in the UK [10]. If proven, this may lead to a truly cost-effective vaccine-booster combination.

MVC-COV1901 is a subunit vaccine based on the stable prefusion spike protein (S-2P) of SARS-CoV-2 adjuvanted with CpG 1018 and aluminum hydroxide and has been approved for use after a large phase 2 clinical trial demonstrated a favorable safety and immunogenicity profiles [11]. An EUA was granted to MVC-COV1901 in July 2021, and the vaccine was rolled out in Taiwan since the end of August 2021. Based on a post-marketing safety surveillance system run by the Taiwanese Centers for Disease Control, no alarming safety signals have been reported for MVC-COV1901 [12]. Thus, we are investigating if MVC-COV1901 boosters can attain optimal immunogenicity after waning immunity from initial immunization(s).

In this study, we report results from an MVC-COV1901 booster shot after two initial immunizations with AZD1222. We used data to quantify anti-SARS-CoV-2 spike IgG antibody titers and neutralizing antibody titers boosted by an MVC-COV1901 booster dose and describe the initial safety findings of the booster dose. We also investigated the enhancement in immunogenicity of a booster dose of MVC-COV1901 against the Omicron variant pseudovirus.

## 2. Methods

### 2.1. Study Design

**Figure 1:**
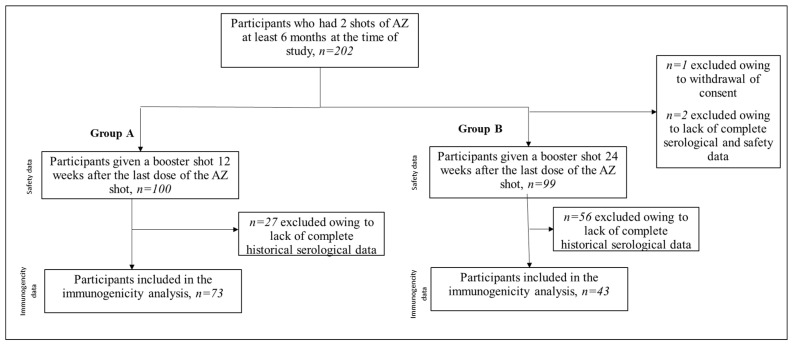
Study design and flowchart.

**Figure 2:**
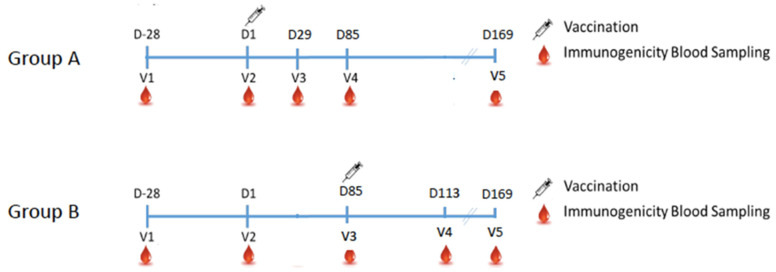
Study timeline and key time points.

This study is a parallel, prospective, randomized, open-label clinical study to evaluate the immunogenicity, safety, and tolerability of MVC-COV1901 as a booster vaccine in participants that have received two doses of AZD1222. It was conducted from 7 October 2021 to 22 April 2022, during which a global transition of the most prevalent strain from Delta to Omicron was observed [13]. In this case, 201 healthy adults aged 23 to 66 years who received two doses of AZD1222 within 6 months of study initiation were randomized into two groups. Participants in Group A were scheduled to receive a booster dose of MVC-COV1901 (15 mcg of S2-P adjuvanted with 750 mcg of CpG 1018 and 375 mcg of aluminum hydroxide) administered intramuscularly 12 weeks after the last dose of AZD1222, whereas those in Group B were scheduled to receive a booster dose of MVC-COV1901 administered intramuscularly 24 weeks after the last dose of AZD1222. For analysis of the immunogenicity, 73 participants for Group A and 43 participants for Group B were included. The study design and flowchart are presented in Figure 1. The timeline of the study is outlined in Figure 2. Day 1 is the day that Group A participants received the dose of MVC-COV1901 booster. Therefore, in Group B participants, the MVC-COV1901 is administered on Day 85. Blood samples were taken at immunization and during additional study visits (Days 29, 85 and 169). At the time the study was conducted, there were no endemic cases of COVID-19 in the community. Enrolled participants have no previous history of COVID-19 infection. Based on PCR results, no breakthrough cases have occurred throughout the course of the trial.

### 2.2. Outcomes

Safety was assessed by monitoring for solicited and unsolicited adverse events (AEs) for the first 14 days after the booster dose, and 28 days and 12 weeks after the booster dose. Immunogenicity was assessed by measuring anti-SARS-CoV-2 spike IgG antibody and live-SARS-CoV-2 neutralizing assay against the ancestral strain as previously performed [11]. Additionally, a pseudovirus neutralization assay was also conducted against the Omicron variant. An electronic case report form (eCRF) was used to record the actual date and time of sample collection. Unique sample identification was used to maintain blinding at the laboratory and allow for automated sample tracking and storage.

### 2.3. Laboratory Methods

The detection and characterization of antigen-specific immunoglobulin were performed by a central laboratory using a validated enzyme-linked immunosorbent assay (ELISA) method using customized 96-well plates coated with S-2P antigen, as previously described [11]. The GMT of the anti-spike IgG titer for NIBSC 20/136 was 109,609, which was calculated from seven repeated tests. The NIBSC 20/136 was assigned as 1000 BAU/mL, and from this, a conversion factor of 0.0912 (1000/109,609) was derived to estimate the BAU/mL values from antigen-specific immunoglobulin titer.

Live-SARS-CoV-2 neutralization assay was performed as previously with ancestral strain SARS-CoV-2, Taiwan CDC strain number 4 (hCoV-19/Taiwan/4/2020; GISAID accession ID EPI_ISL_411927) [11]. The serum samples underwent a total of eight two-fold dilutions, starting from a 1:8 dilution to a final dilution of 1:1024. Diluted serum samples were then mixed with an equal volume of 100 TCID_50_ per 50 μL of virus and incubated at 37 °C for 1 h. After incubation, the mixture was added to Vero E6 cells and incubated at 37 °C in a 5% CO_2_ incubator for 4–5 days. The neutralizing titer (NT_50_) was estimated as the reciprocal of the highest dilution capable of inhibiting 50% of the cytopathic effect. The NT_50_ results were calculated with the Reed-Muench method. Anti-spike IgG titers and neutralizing antibody titers were converted to the WHO Standardized Unit, BAU/mL and IU/mL, respectively. The conversion is based on the WHO-validated NIBSC reference panel. The results are expressed as a geometric mean titer (GMT) and converted to binding antibody units (BAU/mL) for IgG titer and international units (IU/mL) for neutralizing antibody titer as we have performed in our phase 2 clinical study [11].

Pseudoviruses with spike proteins of ancestral strain and Omicron variant (BA.1) were constructed and neutralization assays performed. Two-fold serial dilution of serum samples was mixed with an equal volume of pseudovirus and incubated at 37 °C for 1 h before adding to the HEK-293-hAce2 cells. In this case, 50% inhibition dilution titers (ID_50_) were calculated with uninfected cells as 100% neutralization and cells transduced with the virus as 0% neutralization. The mutations for the Omicron variant used in the spike sequence for pseudovirus construction are A67V, del69-70, T95I, G142D, del143-145, del211, L212I, ins214EPE, G339D, S371L, S373P, S375F, S477N, T478K, E484A. Q493R, G496S, Q498R, N501Y, Y505H, T547K, D614G, H655Y, N679K, P681H, D796Y, N856K, Q954H, N969K, L981F.

## 3. Results

A total of 202 participants were initially recruited. Figure 1 shows the study flowchart and subsets used for the safety and immunogenicity analyses. The demographic characteristics of the population are summarized in Table 1. As expected from the randomization process, Group A and B have similar properties. The median age for both groups is 40 years with an Interquartile range (IQR) of 13.0 and 18.0 years, respectively, for Group A and B. Approximately 32% and 29% are males and females, respectively. In terms of Body Mass Index (BMI), both groups have approximately 13% of the sample BMI greater than 30 kg/m^2^.

Injection of the third shot of MVC- COV1901 in both groups was generally safe and no adverse effects greater than mild (grade 1) to moderate (grade 2) were reported. No serious adverse event was reported after a booster shot with MVC-COV1901 in participants who had received two immunizations with AZD1222. Administration of a booster shot of MVC-COV1901 24 weeks after the last AZD1222 dose was also shown to have favorable safety, reactogenicity and tolerability with slightly fewer adverse events than in those receiving the vaccine 12 weeks after the second AZD1222 dose. Reported adverse events were also mostly mild with some moderate events. Table 2 presents the safety profile of the third dose of MVC-COV1901. Among recipients of the MVC-COV1901 booster shot, approximately 72% and 69% experienced pain or tenderness at the site of injection within 14 days after the shot for groups A and B, respectively. A noticeably lower proportion (i.e., 46% for group A and 26% for group B) of participants reported injection-site induration or swelling. The majority of those who encountered local solicited side effects reported grade 1 or mild effects while a small percentage experienced moderate reactions. In terms of systemic solicited AE, 51% (in group A) and 38% (in group B) experienced malaise or fatigue while none experienced fever. The most commonly reported systemic events in Group A, were malaise or fatigue and myalgia (51% and 39%, respectively). Similarly, malaise or fatigue (38%) and myalgia (36%) were also the most common systemic events in Group B. Most systemic reactions reported were grade 1 with a few grade 2 reactions.

**Figure 3:**
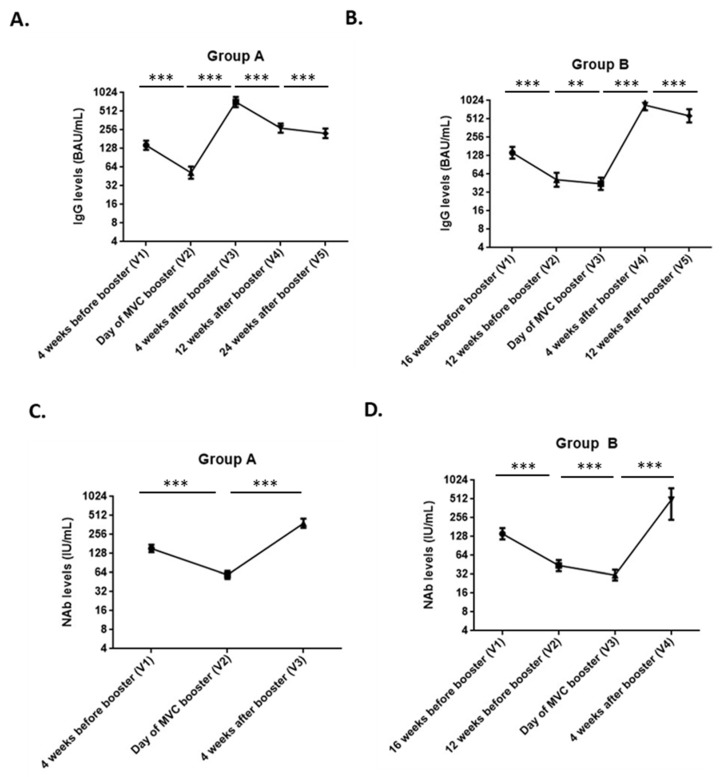
Anti-SARS-CoV-2 spike IgG titers (in BAU/mL) and neutralizing antibody (nAb) titers (in IU/mL) at different time points for both groups A and B. The results were presented by line graph with bars representing geometric mean IgG and nAb titers with error bars for 95% confidence interval values. The following graphs are featured in the figure (**A**) IgG titers in BAU/mL for group A from V1 to V5; (**B**) IgG titers in BAU/mL for group B from V1 to V5; (**C**) nAb titers in IU/mL for group A at different time points; and (**D**) nAb titers in IU/mL for group B at different time points. Comparisons between two consecutive time points were made using Wilcoxon signed-rank test (** *p* < *0*.05, *** *p* < 0.01).

The results of immunogenicity are summarized in Table 3 and Figure 3. Figure 3A,B show the anti-spike IgG titers while Figure 3C,D show neutralizing antibody titers at different time points. For the illustration of anti-spike IgG levels among individuals in Group A, V1: 4 weeks before booster; V2: day of vaccination for the MVC-COV1901 booster shot within three months after the last AZD1222 dose; V3: four weeks after a booster dose of MVC-COV1901; V4: 12 weeks after a booster dose of MVC-COV1901 and V5: 24 weeks after a booster dose of MVC-COV1901. Figure 3A shows that at V1, the anti-spike IgG geometric mean was 144.7 BAU/mL and the levels dropped to 52.1 BAU/mL at V2. At one month after the MVC-COV1901 booster dose (V3), anti-spike IgG levels increased to 724.9 BAU/mL or an almost 14-fold increase compared to that of the day of booster dose (V2). Compared to V1, boosted by the third dose of MVC-COV1901 elicited a 5.0-fold increase in anti-spike IgG immune response. At V4, the anti-spike IgG levels started to decrease slightly with a 2.6-fold decrease compared to V3, while at V5, the anti-spike IgG geometric mean titer (GMT) was reduced to 3.2 times its levels in V3. For group B (Figure 3B), i.e., those who received a delayed booster dose of MVC-COV1901, Figure 3C shows that anti-spike IgG GMT was 142.3 BAU/mL at V1 (28 days after the last shot in the AZD1222 series or 4 weeks before the booster shot). It decreased to 52.9 BAU/mL at V2 (12 weeks before the booster) and further dropped to 44.4 BAU/mL at V3 (the day of the MVC-COV1901 booster). Administration of MVC-COV1901 as a booster greatly increased IgG titers which peaked at V4 (4 weeks after the booster) to 866.8 BAU/mL or almost 20 times its levels at V3. By V5 (12 weeks after booster), anti-spike IgG GMTs started to decline with levels at 576.6 BAU/mL, equating to a 1.5-fold reduction when compared to V4.

As shown in Table 3, the neutralizing antibody (NAb) titer for group A increased from 59.0 IU/mL at the time of the booster dose to 385.4 IU/mL one month after the MVC-COV1901 booster dose. This amounted to a 6.5-fold increase in NAb levels at V3 compared to V2 in terms of IU/mL or an 8.6-fold increase in NT_50_. As in anti-spike IgG titers, fold-increase in NAb titers after the booster dose was also higher in group B. At one month after the booster dose, levels rose to 14-fold of pre-booster NAb levels in IU/mL and a 20-fold increase in NT_50_ levels.

**Figure 4:**
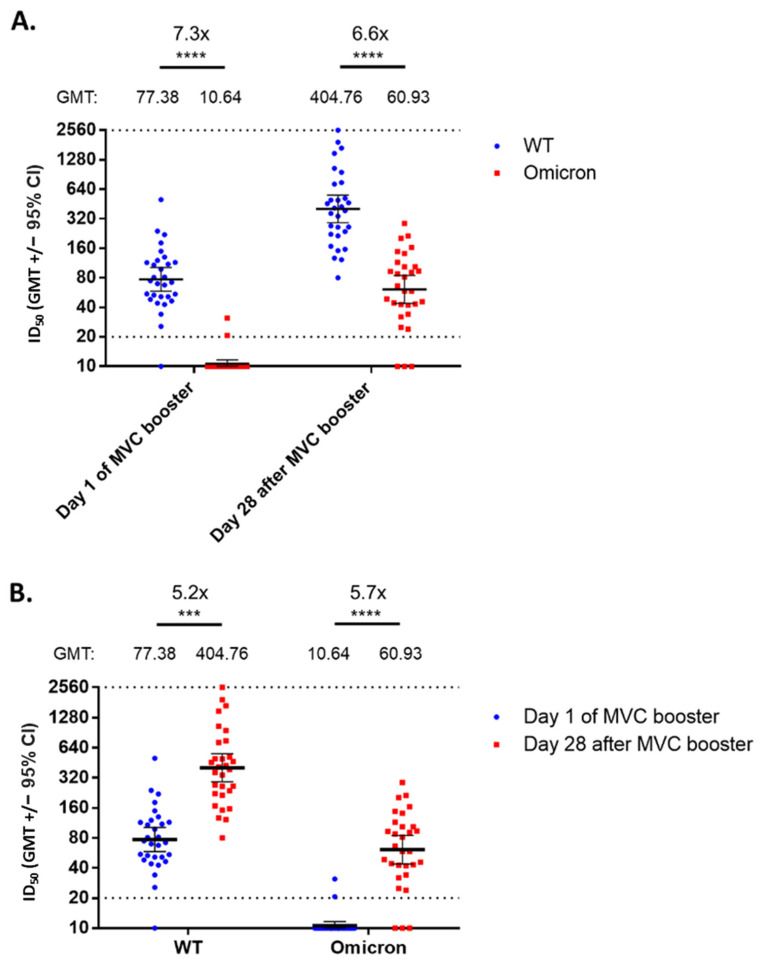
Neutralizing assay against ancestral strain and Omicron variant pseudoviruses. Serum samples on the day of the MVC booster dose and day 28 after the MVC booster dose from 30 randomly selected individuals of the immunogenicity analysis subset from group A (i.e., MVC−COV1901 booster administered within 12 weeks from the last AZD1222 dose) were taken. (**A**) grouped according to the time of sampling; (**B**) grouped according to pseudovirus type. The results were presented by horizontal bars representing geometric mean titer with error bars for 95% confidence interval values. Statistical significance was calculated with the Kruskal-Wallis test with corrected Dunn’s multiple comparisons test. *** = *p* < 0.001, **** = *p* < 0.0001.

To test the neutralizing ability against the Omicron variant by antibodies induced by the MVC-COV1901 booster, we randomly selected pre- and post-booster serum samples from 30 participants of those who were vaccinated within 12 weeks after the last AZD1222 dose and subjected them to neutralizing assay against ancestral strain and Omicron variant pseudoviruses. Before the booster, two doses of AZD1222 were largely ineffective in neutralizing the Omicron variant pseudovirus at a 7.3-fold reduction in GMT compared to the ancestral strain, with only two individuals having detectable levels of neutralizing antibodies (Figure 4A). After the booster, about 90% of the individuals had detectable neutralizing titers against the Omicron variant pseudovirus. Compared to pre-booster levels, there were 5.2- and 5.7-fold increases in GMT levels against the ancestral strain and Omicron variant pseudoviruses compared to pre-booster levels, respectively (Figure 4B).

## 4. Discussion

The antibody quantifications presented here demonstrate that following a waning period post second immunization with AZD1222, a booster shot with MVC-COV19 significantly raises binding antibody levels by as much as 14-fold. More importantly, the maximum antibody levels achieved with the booster exceed those achieved by two immunizations with AZD1222. Similar results have been observed in an extension of the MVC-COV1901 phase 1 trial that followed the same observation and vaccination time points as this study but used MVC-COV1901 for all three immunizations [14,15].

The aggregated results from the phase 1 study and the study presented here suggest that options of a booster vaccine may not be limited to the matching vaccines for the primary series. More importantly, boosters may have the general property of efficiently raising neutralizing antibodies to levels not achieved by prior immunizations [16,17]. Based on the correlates of protection published from Phase III data of AZD1222, the predicted vaccine efficacy against symptomatic infection against the ancestral strain at four months after the second AZD1222 dose was below 60%, and after the booster the predicted VE was raised to 89.7%. [18]. There is, however, an important follow-up question that is to determine if the neutralizing antibody levels are better maintained long-term after one or more boosters. The recent surge of infections driven by a new VoC, the Omicron variant, was alarming in regards to the fact that two doses of the currently-available vaccines became largely ineffective [4,18]. As more data came to light on the effectiveness of booster doses to improve neutralizing against the Omicron variant and other VoCs, booster doses remain one of the few viable options to mitigate the waves of infections [19,20,21]. In this study, we have shown that a booster dose of MVC-COV1901 after two doses of AZD1222 can result in similar increases in neutralizing titer levels against the ancestral strain and Omicron variant pseudoviruses (Figure 4). Administration of booster dose restored the neutralizing ability against the Omicron variant that was previously undetectable with only two doses of vaccination, as in line with other booster studies [18,19,20]. Recent real-world results of three doses of mRNA-1273 against the VoCs showed that the vaccine efficacies against Delta and Omicron infection were 95.2% and 62.5%, respectively [18]. The immunogenic response and favorable safety profile of the third booster shot might offer a viable option for vaccination program designers to cope with the issue of waning immunity after the two-dose schedule compounded by the emergence of variants of concern. The outcomes of the MVC-COV1901 booster remain to be seen in the real-world efficacy study.

A recent study in the UK for heterologous third dose booster regimen (COV-BOOST) showed that at one month after the booster dose compared to the day of the booster dose; there was a 2.6-fold increase in anti-spike IgG titer with two doses plus a booster dose of AZD1222 and a 6.7-fold increase with two doses of AZD1222 and a booster dose of Novavax NVX-CoV2373, also a protein subunit vaccine [10]. In our study, a booster dose of MVC-COV1901 after two doses of AZD1222 showed a 14-fold increase in anti-spike IgG titer, superior to that of the above two combinations and nearly equal to that of boosting with BNT162b2 after two doses of AZD1222, which achieved a 16-fold increase in the COV-BOOST study [10]. In terms of reactogenicity, we have observed no occurrence of severe (grade 3) adverse events after booster administration, while severe solicited reactions have been reported for all of the heterologous booster combinations in the COV-BOOST study [10]. Most noticeably, no incidence of fever has been reported in this study, similar to our previous clinical findings [11].

The study did not investigate T cell responses. However, although not analyzed in the study, the literature points out that many heterologous vaccination schedules produce more immunogenic responses than homologous schedules, but T cell responses depend on the platforms utilized and the sequence of the prime-boost regimen [22]. A study on COVID-19 vaccines comparing a homologous schedule of an inactivated vaccine (I-I-I) and a heterologous schedule with a recombinant subunit vaccine (I-I-S) reveal that the two strategies differed significantly not only in the induction of neutralizing antibodies but also in the composite pattern of neutralizing antibodies and the population of virus-specific CD4+ T cells produced [23]. To date, there is limited evidence on the T cell responses of a protein subunit booster to a primary regimen of an adenoviral vector vaccine among COVID-19 vaccines. Evidence suggests that a single dose of AZD1222 induces a Th-1 biased response characterized by interferon- γ and tumor necrosis factor-α cytokine secretion by CD4+ T cells together with antibody production predominantly of IgG1 and IgG3 subclasses [24]. Similarly, two doses of MVC-COV1901 have a Th1-skewed immune response marked by the induction of substantially high numbers of interferon- γ producing cells [25]. Lorin et al. [26], in an animal study of an HIV vaccine, found that a prime-boost regimen involving a non-human adenoviral vector and an adjuvanted F4 protein elicits good T cell responses in macaques. When administered in a heterologous schedule, both candidate vaccines complemented each other and induced potent and persistent peripheral blood HIV-1-specific CD4+ and CD8+ T cell responses. This provides evidence that a heterologous schedule of an adjuvanted protein vaccine and non-human adenoviral vector vaccine may be a feasible and attractive strategy for immunization.

For vaccines that confer transient protection, the timing of a booster dose remains a crucial component of vaccination programs and could affect the long-term dynamics of disease and immunity [26]. A longer interval between the prime series and booster has been found to increase immunogenicity in general [27,28,29]. Even so, the optimal scheduling of booster doses remains unclear and undetermined. Findings of the study indicate that administration of MVC-COV1901 after 6 months of completion of an adenoviral vector vaccine series is still optimal in terms of immunogenicity and safety. However, the decrease in neutralizing antibodies between the two different schedules should be noted, especially when deciding on the optimal booster schedule. Additionally, there is the question of lagging vaccine coverage and delayed administration of booster doses in LMIC [30]. Current problems such as supply constraints and threats to vaccine equity form the crux of these issues, which, when unaddressed, pose a significant risk of infection and severe disease in different populations. The results of this study bring important implications on issues surrounding vaccine access among LMICs. They can provide policymakers in LMICs with evidence and options in vaccine scheduling to sustain protection in the population.

The limitations of the study include the small sample size used to analyze historical immunogenicity data. Comparison of differences was, however, made through non-parametric statistics. Furthermore, the study did not analyze cell-mediated immunity and T cell responses. Additionally, a pseudovirus neutralization assay was used to test against the Omicron variant. This may not accurately reflect the neutralizing ability against the Omicron variant.

## 5. Conclusions

Our findings showed that administration of MVC-COV1901 as a third dose booster could effectively and safely enhance immunogenicity nearly to the level seen in using mRNA vaccine as a booster dose Our strategy could be aptly applied to lower- and middle-income countries with difficulties in handling mRNA booster vaccines but desirable to achieve a similarly high level of immunity against SARS-CoV-2.

## Figures and Tables

**Table 1:** Demographic profile of respondents.

Item	Vaccine Group		*p* Value
	Group A	Group B	
Age (years)			
*n* (Missing)	100 (0)	99 (0)	0.763
Mean (SD)	41.4 (10.3)	41.8 (10.8)	
Median (IQR)	40 (13.0)	40 (18.0)	
Q1–Q3	34.5–47.5	33–51	
Min–Max	24–66	23–64	
Gender			
*n* (Missing)	100 (0)	99 (0)	0.679
Male	32 (32.0)	29 (29.3)	
Female	68 (68.0)	70 (70.7)	
BMI (kg/m^2^)			
*n* (Missing)	100 (0)	99 (0)	0.836
Mean (SD)	24.4 (4.4)	24.6 (4.2)	
Median (IQR)	23.97 (6.03)	23.8 (6.3)	
Q1–Q3	21.2–27.2	21.3–27.6	
Min–Max	17.6–35.3	18.0–37.8	
BMI group			
*n* (Missing)	100 (0)	99 (0)	0.978
<30 kg/m^2^	87 (87.0)	86 (86.9)	
≥30 kg/m^2^	13 (13.0)	13 (13.1)	
Comorbidity Category			
*n* (Missing)	100 (0)	99 (0)	0.224
Yes	32 (32.0)	24 (24.2)	
No	68 (68.0)	75 (75.8)	

Abbreviations: *n*= number of subjects in PPI population; SD = standard deviation; Q1 = first quartile (25th percentile); Q3 = third quartile (75th percentile); IQR = interquartile range; BMI = Body Mass Index.

**Table 2:** Solicited Adverse Effects 14 days after an MVC-COV1901 booster shot.

Item	Group A			Group B		
	Event	Subject	Percentage	Event	Subject	Percentage
N (missing)	100 (0)			99 (0)		
At least one event	257	82	82%	208	76	78%
Local	120	74	74%	94	70	71%
Pain/Tenderness	72	72	72%	68	68	69%
Grade 1	71	71	71%	68	68	69%
Grade 2	1	1	1%	0	0	0%
Erythema/Redness	2	2	2%	0	0	0%
Grade 1	2	2	2%	0	0	0%
Grade 2	0	0	0%	0	0	0%
Induration/Swelling	46	46	46%	26	26	26%
Grade 1	46	46	46%	26	26	26%
Grade 2	0	0	0%	0	0	0%
Systemic	137	63	63%	114	54	55%
Malaise/Fatigue	51	51	51%	38	38	38%
Grade 1	48	48	48%	32	32	32%
Grade 2	3	3	3%	6	6	6%
Myalgia	39	39	39%	36	36	36%
Grade 1	35	35	35%	31	31	31%
Grade 2	4	4	4%	5	5	5%
Headache	30	30	30%	17	17	17%
Grade 1	29	29	29%	15	15	15%
Grade 2	1	1	1%	2	2	2%
Diarrhea	7	7	7%	12	12	12%
Grade 1	7	7	7%	11	11	11%
Grade 2	0	0	0%	0	0	0%
Nausea/Vomiting	10	10	10%	11	11	11%
Grade 1	10	10	10%	8	8	8%
Grade 2	0	0	0%	3	3	3%
Fever	0	0	0%	0	0	0%
Grade 1	0	0	0%	0	0	0%
Grade 2	0	0	0%	0	0	0%

**Table 3:** Immunogenicity on the day of MVC-COV1901 booster (V2 for Group A, V3 for Group B) and one month after MVC-COV1901 booster (V3 for Group A, V4 for Group B) as measured by anti-SARS-CoV-2 spike IgG titers and live virus neutralizing antibody titers. GMTs are shown as GMT with a 95% confidence interval in the parentheses. Fold changes are calculated as the geometric mean of the V3/V2 ratio (for Group A) or V4/V3 ratio (for Group B) of individual titer values with a 95% confidence interval in the parentheses.

Group A	Unit	V2 (*n* = 73)	V3 (*n* = 73)	Fold Change V3/V2	* *p*-Value
Anti-SARS-CoV-2 spike IgG	IgG GMT	571.4 (456.6–715.1)	7948.7 (6558.5–9633.6)	13.9 (10.5–18.4)	<0.0001
	BAU/mL GMT	52.1 (41.6–65.2)	724.9 (598.1–878.6)	13.9 (10.5–18.4)	<0.0001
Neutralizing antibody	NT_50_ GMT	66.6 (56.6–78.4)	569.7 (471.7–688.0)	8.6 (7.0–10.5)	<0.0001
	IU/mL GMT	59.0 (51.2–68.0)	385.4 (326.8–454.5)	6.5 (5.5–7.8)	<0.0001
Group B	Unit	V3 (*n* = 43)	V4 (*n* = 43)	Fold change V4/V3	*p*-value
Anti-SARS-CoV-2 spike IgG	IgG GMT	487.4 (384.9–617.0)	9504.1 (7817.9–11,554.0)	19.5 (14.4–26.4)	<0.0001
	BAU/mL GMT	44.4 (35.1–56.3)	866.8 (713.0–1053.7)	19.5 (14.4–26.4)	<0.0001
Neutralizing antibody	NT_50_ GMT	31.9 (25.4–40.1)	651.9 (535.1–794.2)	20.4 (15.2–27.5)	<0.0001
	IU/mL GMT	31.0 (25.4–37.8)	433.6 (364.9–515.4)	14.0 (10.8–18.1)	<0.0001

* Using Wilcoxon signed rank test.

## Data Availability

The original data presented in the study are available upon reasonable request to the authors.
